# Text mining approaches for dealing with the rapidly expanding literature on COVID-19

**DOI:** 10.1093/bib/bbaa296

**Published:** 2020-12-07

**Authors:** Lucy Lu Wang, Kyle Lo

**Affiliations:** The Allen Institute for Artificial Intelligence, Seattle, WA 98112, USA; The Allen Institute for Artificial Intelligence, Seattle, WA 98112, USA

**Keywords:** COVID-19, text mining, natural language processing, information retrieval, information extraction, question answering, summarization, shared tasks, CORD-19

## Abstract

More than 50 000 papers have been published about COVID-19 since the beginning of 2020 and several hundred new papers continue to be published every day. This incredible rate of scientific productivity leads to information overload, making it difficult for researchers, clinicians and public health officials to keep up with the latest findings. Automated text mining techniques for searching, reading and summarizing papers are helpful for addressing information overload. In this review, we describe the many resources that have been introduced to support text mining applications over the COVID-19 literature; specifically, we discuss the corpora, modeling resources, systems and shared tasks that have been introduced for COVID-19. We compile a list of 39 systems that provide functionality such as search, discovery, visualization and summarization over the COVID-19 literature. For each system, we provide a qualitative description and assessment of the system’s performance, unique data or user interface features and modeling decisions. Many systems focus on search and discovery, though several systems provide novel features, such as the ability to summarize findings over multiple documents or linking between scientific articles and clinical trials. We also describe the public corpora, models and shared tasks that have been introduced to help reduce repeated effort among community members; some of these resources (especially shared tasks) can provide a basis for comparing the performance of different systems. Finally, we summarize promising results and open challenges for text mining the COVID-19 literature.

## Introduction

Since the discovery of the novel coronavirus SARS-CoV-2 [4, 107] toward the tail end of 2019, the disease caused by the virus, COVID-19, has swept through the globe and drastically altered all aspects of our lives. Governments and researchers, academic and industry alike, have coalesced around the common goals of healthcare resource management, social policy determination, prevention and treatment and vaccine development. The scientific community, correspondingly, has responded rapidly to the pandemic. Scientific output on the subject of COVID-19 and coronaviruses has emerged at an unprecedented rate, placing significant strain upon clinicians, researchers and others who must keep up-to-date on this new literature. By different metrics, somewhere upwards of 55–100 000 papers and preprints on COVID-19 have been released in 2020 thus far (please refer to https://www.ncbi.nlm.nih.gov/pmc/about/covid-19/, https://www.semanticscholar.org/cord19 and https://covid19primer.com/dashboard for possible paper counts; estimate made on 12 September 2020), accelerating to the current rates of many hundreds of new articles a day. Even on the low-end of this estimate, conventional reading methods are challenged and we must rely on automated text mining approaches to address this tidal wave of research output.

One of the major application areas of biomedical text mining is managing information overload [3, 19, 40, 116]. As per [19], text mining focuses on solving specific problems such as retrieving relevant documents or extracting nuggets of information from those documents. In the process of addressing these problems, text mining systems may use techniques for information retrieval, information extraction, text classification, etc. and leverage methods from related fields such as natural language processing and knowledge base (KB) construction. While there lacks consensus on the precise relationships between these various tasks and/or fields of study [3, 19, 40, 116], in this review, we focus on approaches for addressing information overload and adopt ‘text mining’ as a general term to refer to methods from the aforementioned areas.

In response to the large volume of literature published on COVID-19, the computing community has introduced text mining corpora, modeling resources, systems and community-wide shared tasks specific to COVID-19 to address the mounting challenge. Corpora are collections of documents, preprocessed to extract machine-readable text, that are used for text mining; in this case, we focus on corpora-containing scientific articles. Modeling resources can be incorporated by text mining practitioners into production systems and consist of things such as text embeddings, data annotations, pretrained language models, knowledge graphs and more. Systems are applications that incorporate text mining models and user interfaces to provide functionalities such as the ability to search, discover or visualize article content. Shared tasks are community competitions that promote concentrated work on specific scientific problems.

**Fig. 1:**
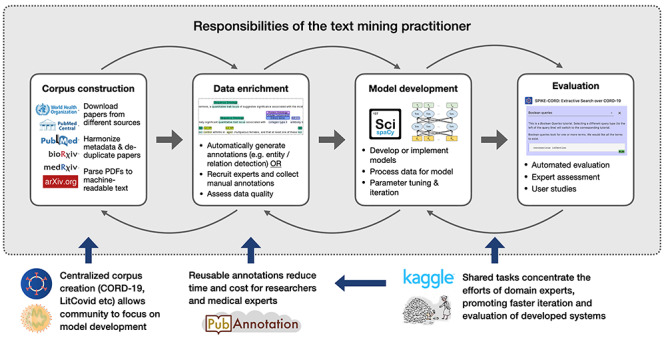
A typical workflow for creating a literature text mining system may consist of corpus construction, data enrichment, model development and evaluation. A text mining practitioner (e.g. engineer, researcher, enthusiast, etc.) may be responsible for each of these steps in the gray box, whether by identifying and adapting existing datasets and models or by creating their own. For COVID-19, centralization of parts of this workflow have helped to reduce the burden around some of these steps.

Figure 1 illustrates how a text mining practitioner might approach developing a system to address information overload for researchers. Unfortunately, the process of corpus construction, data enrichment, model development, evaluation and eventual deployment can take months or years, which is unacceptable during a public health crisis. In the current situation, public corpora help to remove the burden of corpus creation, while shared community annotations contribute to addressing the challenges of data enrichment and annotation. Finally, shared tasks help to promote faster iteration of this process by centralizing evaluation and also serving as a source of annotated data.

In this review, we summarize the corpora (Section on “Text mining corpora”), modeling resources (Section on “Text mining modeling resources”), systems (Section on “Text mining systems”) and shared tasks (Section on “Shared tasks”) that have been created/implemented to support text mining over the COVID-19 literature. We note standout systems that either provide strong performance on fundamental tasks such as search or question answering (QA) or provide novel functionality such as multi-document summarization or linking between articles and clinical trials. We also discuss strategies for building performant and useful systems, specifically advocating for systems that facilitate the production of systematic reviews, or those that directly address the needs of clinicians, researchers and public health officials.

## Text mining corpora

One of the earliest and largest literature corpora created to support COVID-19 text mining is the COVID-19 Open Research Dataset (CORD-19, https://www.semanticscholar.org/cord19) [98], a corpus of metadata and full text of COVID-19 publications and preprints released daily by Semantic Scholar at the Allen Institute for AI, in collaboration with Microsoft Research, IBM Research, Kaggle, Chan-Zuckerberg Initiative, the National Library of Medicine (NLM) at the National Institutes of Health (NIH) and Georgetown’s Center for Security and Emerging Technology. This corpus was first released on 16 March 2020 at the request of The White House Office of Science and Technology Policy, to support community-wide efforts to apply text mining techniques to the coronavirus literature. The corpus combines papers from the PubMed Central (PMC), PubMed, World Health Organization (WHO)’s COVID-19 database (https://www.who.int/emergencies/diseases/novel-coronavirus-2019/global-research-on-novel-coronavirus-2019-ncov) and preprint servers bioRxiv, medRxiv and arXiv. Paper metadata from these sources are harmonized, PDFs are converted into machine-readable JSON using the S2ORC pipeline described in [54] and HTML representations of tables in papers are added using IBM Watson Discovery’s Global Table Extractor [115]. As of 15 September 2020, the corpus contains more than 260 000 paper entries (with 105 000 full text entries). The majority of systems described in Section on “Text mining systems” use this corpus in some way.

LitCovid is a curated set of open access COVID-19 papers from PubMed [16], containing 52 000 papers at the time of writing and growing. Several text mining systems described in Section on “Text mining systems” use LitCovid as a source of data. LitCovid initially provided a much-needed complementary set of papers to CORD-19, since the early releases of CORD-19 focused on PMC, bioRxiv and medRxiv as sources and did not include papers from PubMed. However, the releases of CORD-19 published after 19 May include PubMed as a source of papers.

Other curated sets of COVID-19 papers are also available, e.g. the WHO’s COVID-19 database or the Centers for Disease Control and Prevention (CDC)’s COVID-19 research articles database(https://www.cdc.gov/library/researchguides/2019novelcoronavirus/researcharticles.html). These databases overlap with other corpora; for example, the WHO database is ingested by CORD-19 and much of the CDC database overlaps with PubMed and PMC, also sources of papers in CORD-19 and LitCovid. The CDC database also provides a complementary document collection of white papers and technical reports.

Lastly, several publishers have compiled and released collections of their COVID-19 literature, such as Elsevier’s Novel Coronavirus Information Center (https://www.elsevier.com/connect/coronavirus-information-center), Springer Nature’s Coronavirus Research Highlights (https://www.springernature.com/gp/researchers/campaigns/coronavirus), JAMA Network’s COVID-19 Collection (https://jamanetwork.com/journals/jama/pages/coronavirus-alert) or Science’s COVID-19 Collection (https://www.sciencemag.org/collections/coronavirus). Many publishers have provided COVID-19 literature under temporary open access licenses through PMC’s Public Health Emergency COVID-19 Initiative (https://www.ncbi.nlm.nih.gov/pmc/about/covid-19/), thus making these texts available to the public through PMC and through aggregate corpora like CORD-19 (though we emphasize that this does not include all publishers, e.g. JAMA does not, which limits the community’s ability to create a truly comprehensive corpus). Full text may also be unavailable in some cases or may only be available in the form of PDFs, which must undergo extensive preprocessing to extract full text for text mining. Finally, the open access status for many articles is under-specified, which could result in unpleasant revocation of licenses and nullification of datasets and systems in the future.

## Text mining modeling resources

We describe modeling resources that are primarily used to support downstream text mining applications. These resources include paper and concept embeddings, reusable text annotations, knowledge graphs or domain-adapted contextual language models. An overview of these resources is provided in Table 1. In this table, we provide the data and models used in the creation of each resource, as well as a short description of the resource, which can help guide its use in downstream text mining systems.

**Table 1:** Resources for text mining researchers and practitioners, including embeddings, reusable annotated datasets, knowledge graphs and pretrained COVID-19-domain language models. These resources can be incorporated into downstream systems. Under ‘Affiliation’, we use $^\dagger $ for industry, $^*$ for nonprofit and no symbol for academic affiliations; if no affiliation is provided, the work is conducted by independent researchers

**Resource type**	**Resource name**	**Data/model used**	**Affiliation**	**Link**	**Description**
Embeddings	SPECTER CORD-19 embeddings	CORD-19	Allen Institute for AI$^*$	https://www.semanticscholar.org/cord19	SPECTER embeddings [19] for CORD-19 papers
	COVID-19 Concept embeddings	CORD-19, SNOMED-CT	The Ohio State University	https://slate.cse.ohio-state.edu/JET/COVID-19/	JET embeddings [60] for clinical entities (SNOMED-CT) in CORD-19 corpus
	CORD-19 SeVeN embeddings	CORD-19	Cardiff University	https://github.com/luisespinosaanke/cord-19-seven	SeVeN [28] word embeddings trained on CORD-19
	Co-occurrence network embeddings [63]	CORD-19-on-FHIR	Mayo Clinic	https://github.com/shenfc/COVID-19-network-embeddings	Network co-occurrence embeddings trained on semantically annotated version of CORD-19 (CORD-19-on-FHIR)
Annotations	CODA-19 [35]	CORD-19	Penn State University, UCSF, Carnegie Mellon University	https://github.com/windx0303/CODA-19	Crowdsourced dataset of research aspect annotations for papers in CORD-19
	CORD-19-on-FHIR	CORD-19, FHIR	Mayo Clinic	https://github.com/fhircat/CORD-19-on-FHIR	FHIR RDF version of CORD-19 with annotations of condition, medication, and procedure clinical entities
	COVID-19 DistillerSR	CORD-19, ClinicalTrials.gov	Evidence Partners$^\dagger $	https://www.evidencepartners.com/resources/covid-19-resources/	Links between clinical trial identifiers and documents in CORD-19
	SciBite COVID-19 annotations	CORD-19	SciBite$^\dagger $	https://github.com/SciBiteLabs/CORD19	Sentence and entity co-occurrence annotations; annotation of entities from MeSH, GO, HPO, HGNC, ChEMBL, and more
Knowledge graph	CovidGraph	CORD-19, Lens, Ensembl, NCBI Gene, Gene Ontology, experimental data, Johns Hopkins 2019-nCoV dataset	Many academic and industry organizations	https://covidgraph.org/	A knowledgegraph of COVID-19 papers, case statistics, genes and functions, and molecular data
	KGTK COVID-19 KnowledgeGraph [36]	CORD-19, WikiData, CTD, Blender Lab COVID-KG	USC, Pontificia Universidade Cat’olica Rio de Janeiro	–	A knowledge graph that integrates the CORD-19 corpus with gene, chemical, disease and taxonomic information from Wikidata and CTD databases and the Blender Lab COVID-KG (http://blender.cs.illinois.edu/covid19/)
	Blender Lab COVID-KG [99]	CORD-19	UIUC	http://blender.cs.illinois.edu/covid19/	Knowledge graph with entity types genes, diseases, chemicals and organisms and subtypes derived from the text and figure/caption relations in literature
	COVID-19 KnowledgeGraph [105]	CORD-19, Comprehend Medical [8]	Amazon Web Services (AWS)	https://aws.amazon.com/cn/covid-19-data-lake/	COVID-19 specific knowledge graph; graph embeddings are used to power AWS CORD-19 search
	COVID-KOP [44]	ROBOKOP, GO annotations, SciBite CORD-19 annotations	UNC Chapel Hill	https://covidkop.renci.org/	Combines ROBOKOP biomedical knowledge graph with information extracted from SciBite CORD-19 annotations
Language model	CovidBERT	CORD-19, BioBERT, ClinicalBERT	–	https://github.com/manueltonneau/covid-berts	BioBERT [49] and ClinicalBERT [2] fine-tuned on CORD-19
	GreenCovidSQuADBERT [68]	CORD-19, Word2vec, SQuADBERT	LMU Munich, Siemens$^\dagger $	–	A cheap and performant way to achieve domain adaptation for BERT models; achieves by training Word2vec and aligning Word2vec embeddings to BERT wordpieces

SeVeN indicates semantic vector networks; JET, jointly embedding entities and text; CTD, Comparative Toxicogenomics database.

**Embeddings** are computed vector representations of spans of text that capture semantic and syntactic similarities between these texts. Embeddings can be computed at different levels of granularity, for word tokens, named entities, sentences, paragraphs, documents and beyond. There are dozens of different embedding methodologies; for more information on their use, see [13, 76].

Paper and concept embeddings have been used by several systems to support search and retrieval over the COVID-19 literature. The SPECTER embedding method computes paper embeddings using a SciBERT model [6] pretrained on relatedness signals derived from the citation graph [18]. SPECTER paper embeddings have been shown to successfully capture paper similarity [18] and are available for all papers in CORD-19. Also available for papers in CORD-19 are clinical concept embeddings trained using the JET algorithm [60], relation embeddings trained using SeVeN [28] and network co-occurrence embeddings [63] for biomedical entities computed using CORD-19-on-FHIR. Embeddings capture text similarity and can be used to retrieve similar texts, e.g. the embedding of a query text can be used to retrieve relevant documents from the same embedding space. 

**Annotations** provide information in addition to the metadata and text of the COVID-19 literature. For example, one may wish to identify and annotate mentions of biomedical or clinical entities, relations or other attributes of interest in the paper text. Annotations can be generated automatically (e.g. using pretrained models for named entity recognition and KB entity linking, with tools such as MetaMap Lite [23] or ScispaCy [59]) or manually through expert annotation (e.g. asking a human to label spans describing population, intervention, comparator and outcome (PICO) elements in clinical trial papers). Several groups have published reusable annotations, either independently or through annotation sharing platforms such as PubTator(https://www.ncbi.nlm.nih.gov/research/pubtator/) or PubAnnotation (http://pubannotation.org/). On PubAnnotation, for example, automatically generated annotations of terms from several ontologies and PICO elements are available for the CORD-19 and LitCovid corpora.

Also available is CORD-19-on-FHIR (https://github.com/fhircat/CORD-19-on-FHIR), a version of CORD-19 with semantic annotations to clinical entities in the categories of condition, medication and procedure. This version can be more easily integrated into clinical workflows or used to supply evidence for clinical decision support. For the CODA-19 annotation project [35], the authors demonstrate the ability to create crowd-sourced annotations for papers in CORD-19. Finally, shared tasks are also a source of expert-generated annotations (e.g. EPIC-QA will produce labeled answer spans and TREC-COVID produces document rankings; see Section on “Shared tasks” for more info) that can be leveraged by text mining practitioners to create more performant systems. 

**Knowledge graphs** provide a model of entities and relationships in a particular domain. These graphs can be used to represent background knowledge and can also be used to infer or discover new relationships through reasoning. Several COVID-19 knowledge graphs have been constructed by combining relations detected in the literature with other ontologies and databases of structured relationships. The CovidGraph(https://covidgraph.org/) is perhaps the largest of these, combining literature, case statistics and genomic and molecular data. Another project, the Knowledge Graph Toolkit [36], integrates the CORD-19 corpus with gene, chemical, disease and taxonomic information from Wikidata (https://www.wikidata.org/) and the Comparative Toxigenomics Database (http://ctdbase.org/), as well as the Blender Lab COVID-KG (http://blender.cs.illinois.edu/covid19/) [99], another COVID-19 knowledge graph focused on drug repurposing. These knowledge graphs are used by several systems in Section on “Text mining systems” to provide entity- or relation-based exploration of the literature or as a way to visualize data. Knowledge graphs can also support automated reasoning and inference and the potential discovery of novel relationships. 

**Language models**, specifically pretrained contextual language models, are ubiquitous in modern text mining systems. These models are the state-of-the-art in natural language processing and have significantly outperformed previous baselines on the full spectrum of language-based tasks [24, 53, 67]. Many projects in Section on “Text mining systems” leverage domain-adapted BERT [24] models such as SciBERT [6] and BioBERT [49], which have been fine-tuned to scientific and biomedical text, respectively. Variants of BERT models [24] fine-tuned on COVID-19 literature are available in the form of BioCovidBERT and ClinicalCovidBERT (https://github.com/manueltonneau/covid-berts). Poerner *et al.* [68] also discuss a domain adaptation technique where word2vec [57] vectors trained in a target domain are used to update wordpiece embeddings in a general domain language model like BERT [24], resulting in a lower cost and less resource-intensive, yet still performant alternative. These pretrained models also provide an alternative means to computing text embeddings and can be leveraged for retrieval or classification in a similar way to the other types of vector embeddings described above.

## Text mining systems

Numerous text mining systems for COVID-19 literature have been released in 2020 thus far. We compile a list of 39 systems in Table 2 (we maintain an up-to-date list of systems on the CORD-19 GitHub page https://github.com/allenai/cord19). These text mining systems are collected through a public form on the CORD-19 website, by searching COVID-19 papers and preprints in the CORD-19 corpus and from social media. We omit systems that appear to index documents using off-the-shelf software (e.g. ElasticSearch) without additional data or methodological extensions or without other obvious distinguishing system features.

**Table 2:** COVID-19 text mining systems, including both production systems and research prototypes, covering a range of text mining tasks. Note that ‘Data’ and ‘Methods/Models’ only include known data sources and modeling/implementation details discussed in the associated documentation of these systems. Under ‘Affiliation’, we use $^\dagger $ for industry, $^*$ for nonprofit and no symbol for academic affiliations; if no affiliation is provided, the work is conducted by independent researchers ‘Search’ - users issue queries to system to find relevant content. ‘Augmented reading’ - system provides interface for reading papers with additional features (e.g. term highlighting). ‘Exploration’ - users use system to explore available content, possibly without specific informational need. Often used to understand the underlying data source. ‘KB construction’ - system constructs a KB using extracted entities and relations to support a system function. ‘Visualization’ - data visualization is component of how user interacts with system. ‘Clinical diagnostic support’ - system assists healthcare providers in disease diagnosis. ‘Question answering’ - system expects a query in question form and directly answers user-written query with an (extracted) answer. ‘Summarization’ - system surfaces automated summaries of paper text. ‘Claim verification’ - system expects a query in claim or assertion form and verifies or refutes it.

	**Tasks**	**System**	**Affiliation**	**Link**	**Data**	**Methods/Models**	**User interface**
S1	Search	Covidex [114]	University of Waterloo and NYU	https://covidex.ai/	CORD-19, ClinicalTrials.gov through TrialStreamer	Retrieves passages using Anserini [109]. Reranking using T5-base model [72] finetuned on biomedical text and trained for ranking on MS MARCO [14].	Supports user-written keyphrase queries. Highlights matched terms in abstract. Toggle for searching different corpora.
S2	Search	COVID papers browser	–	https://github.com/gsarti/covid-papers-browser	CORD-19, SNLI, MultiNLI	Matches queries to papers via pretrained sentence embeddings: SentBERT [74] training procedure with SciBERT [6], BioBERT [49], CovidBERT, and ClinicalCovidBERT. Trained on SNLI [11] and MultiNLI [104] datasets.	Supports interactive querying via command line.
S3	Search	fatcat [Covid-19]	Internet archive$^*$	https://covid19.fatcat.wiki/	CORD-19, WHO, Wanfang, CNKI, Internet Archive	ElasticSearch	Supports user-written keyphrase queries. Highlights matched terms in abstract. Toggle for searching different corpora.
S4	Search	KDCovid	Google$^\dagger $, UMass Amherst, MSR Montreal$^\dagger $, UToronto, CMU	http://kdcovid.nl/	CORD-19	Sentence-based retrieval using a similarity metric derived from BioSentVec [17]. BeFree [12] for entity linking. Genes are linked to UniProt and diseases to MedGen. Relations between genes and diseases from DisGeNET (v6) [30].	Supports user-written keyphrase queries, returns relevant papers. Biomedical entities in abstracts color-coded by type. Entity hyperlinks to associated webpages. Gene-disease relations presented for each paper.
S5	Search	DOC Search	DRE$^\dagger $	https://covid-search.doctorevidence.com/	CORD-19, PubMed, ClinicalTrials.gov, WHO ICTRP, news articles, etc.	–	Supports user-written keyphrase and boolean queries comprised of paper metadata, entities and PICO elements
S6	Search	CoronaSearch	–	https://coronasearch.net/	CORD-19	Embeds documents using Google’s Multilingual Universal Sentence Encoder [110]. Retrieves relevant documents using Facebook’s Faiss library [37]	Supports user-written keyphrase queries, with specific emphasis on multi-lingual queries
S7	Search	DISCOVID.AI	Karlsruhe Institute of Technology	https://discovid.ai/	CORD-19	–	Supports user-written keyphrase queries. Results are linked to associated clinical trials.
S8	Search	Vapur [43]	Bogaziçi University	https://vapur.herokuapp.com/	CORD-19, ChemProt	NER with BERN [41], relation extraction model if BioBERT [49] trained on ChemProt [86],	Supports user-written keyphrase queries or query on chemical/gene/RNA compound identifiers (e.g. ChEBI chemical identifier, HGNC gene name). Results organized by relationships with other potential drug targets and entities of interest.
S9	Search	Covid-19 Search	Microsoft Azure$^\dagger $	https://covid19search.azurewebsites.net/	CORD-19	–	Supports user-written keyphrase or boolean queries. Filter resiults using extracted biomedical entity types (e.g. anatomy, disease, gene, drug, etc). Recommends similar papers.
S10	Search	Research-Covid19.ai	Gowi$^\dagger $	https://research-covid19.ai/	CORD-19	Search using Azure Cognitive Search. Entities extracted and normalized using BERN [41].	Supports user-written keyphrase or boolean queries. Filter results using extracted biomedical entity types (e.g. species, disease, gene, drug).
S11	Search, augmented reading	DeScign COVID-19 Search	DeScign$^\dagger $	http://covid.descign.com/	CORD-19	–	Supports user-written keyphrase queries. Filter results using entities (e.g. viral anatomy, chemicals, diseases, biomolecules, etc.). Supports reading of extracted paper full text with highlighted entities.
S12	Search, augmented reading	COVID-19 Intelligent Insight	Sinequa$^\dagger $	https://covidsearch.sinequa.com/	CORD-19, Elsevier, Clinical trial info from WHO’s ICTRP database, arXiv, bioRxiv, medRxiv, COVID-19 papers from BMJ, Web text from WHO and CDC	–	Supports user-written keyphrase queries. Filter results using facets (e.g. indication, human phenotype). Supports reading of paper PDFs with highlighted entities.
S13	Search, exploration	Covid AI-powered Search	Curiosity$^\dagger $	https://covid.curiosity.ai/	CORD-19, SciHub, UMLS, OntoBee, MAG, etc.	–	Supports user-written keyphrase queries. Filter results by paper topics and extracted disease entities linked to KGs (e.g. UMLS, OntoBee, etc.). Provides access CORD-19 papers via other sources (e.g. SciHub).
S14	Search, exploration	COVID-19 Navigator	IBM Watson$^\dagger $	https://covid-19-navigator.mybluemix.net/	CORD-19, Medline, PubMed Open Access, ClinicalTrials.gov, patents from the US Patent Office, UMLS	–	Supports boolean queries using UMLS concepts and semantic types.
S15	Search, exploration	SPIKE-CORD [88]	Allen Institute for AI$^*$	https://spike.covid-19.apps.allenai.org/search/covid19	CORD-19	Entities and syntax extracted using ScispaCy [59]. Data indexed using Odinson [93]. Support custom query syntax.	Specialized query language supports regex operators (e.g. wildcards, number of matches), matching on entity types, and syntactic patterns (e.g. similar verbs).
S16	Search, exploration	EVIDENCEMINER [101]	UIUC	https://evidenceminer.com/	CORD-19, PubMed, UMLS	Retrieves sentences with similar biomedical entities to the query using distantly supervised NER and OpenIE, details in [100].	Supports user-written keyphrase queries that can take the form of a claim, results ranked by the level of evidence they provide toward the query. Entities are highlighted in results. Filter results by entity type.
S17	Search, exploration, KB construction, visualization	Carnap	Funktor LLC$^\dagger $	https://carnap.ai/	CORD-19	–	Supports entity-based queries with medical terms, returns relationships from an underlying KB, supported by papers. Filter results by entity, relation, domain study, or the strength of relationships.
S18	Search, exploration, KB construction, visualization, clinical diagnostic support	Kahun	Kahun$^\dagger $	https://coronavirus.kahun.com/	CORD-19, SNOMED, LOINC, and other clinical ontologies	–	Supports entity-based queries with clinical entities, returns a graph of clinical relationships related to the query entity and COVID-19, supported by papers.
S19	Search, visualization	COVID-SEE [95]	University of Melbourne, IBM Research$^\dagger $	https://covid-see.com/search	CORD-19, EBM-NLP	Retrieves documents using Covidex API [114], entity tagging using MetaMap [23], PICO element extraction using a BiLSTMCRF model [46] model trained on EBM-NLP [62].	Supports user-written keyphrase queries. Browse search results using a Sankey diagram of PICO extractions.
S20	Search, exploration, visualization	Covidexplorer	IIT Gandhinagar’s Lingo Group	http://covidexplorer.in/	CORD-19, Twitter	Biomedical entities (e.g. proteins, diseases, cell types) extracted using SciBERT [6].	Supports user-written keyphrase queries. Papers are tagged with extracted biomedical entities. Filter results using year or entities. Entity pages show frequent co-mentioned entities and timelines of paper mentions. Visualizes COVID-19 Twitter mention trends.
S21	Search, exploration, visualization	CovidScholar	UC Berkeley	https://www.covidscholar.com/	CORD-19, Elsevier, LitCovid, Lens, Dimensions, human submissions	Adapts the MATSCHOLAR [102] system for identifying relevant papers given entity-centric queries.	Supports user-written keyphrase queries comprised of entities. Filter results on paper facets. Search similar papers. Visualizes word embeddings. Integrates user-submitted data corrections.
S22	Search, claim verification	SciFact: CORD-19 Claim Verification [97]	Allen Institute for AI$^*$	https://scifact.apps.allenai.org/	CORD-19, S2ORC, SciFact	Retrieves documents using Covidex API [114]. Uses RoBERTa [53] for evidence selection, and to classify claim-evidence pairs as Supported/Refuted.	Supports user-written queries that take the form of a scientific claim, returns papers supporting or refuting the claim, along with confidence scores.
S23	Search, QA	CO-Search [29]	Salesforce$^\dagger $	https://sfr-med.com/search	CORD-19, ChemProt	Retrieves documents using an ensemble of SiameseBERT [75], TF-IDF and BM25. Reranking model composed of multi-hop question answering module and multi-paragraph abstractive summarizer.	Supports user-written keyphrase queries or natural questions, returns a ranked list of matching articles with highlighted answer spans.
S24	Search, QA	AWS CORD-19 Search [7]	Amazon Web Services (AWS)$^\dagger $	https://cord19.aws/	CORD-19, Amazon Comprehend Medical	Multilabel topic classification of papers using Amazon Comprehend Medical [8]. Search using Amazon Kendra. Research topics learned using LDA.	Supports user-written natural questions, returns a ranked list of matching articles with highlighted answer spans. Filter results by topic. Recommends similar papers.
S25	Search, QA	covidAsk [48]	DMIS Lab of Korea University	https://covidask.korea.ac.kr/	CORD-19, Natural Questions, SQUAD	BEST [50] for keyword matching. DenSPI [79] for longer questions. BERN [41] for named entity extraction. BioSyn [85] for entity linking to CTD or NCBI. Trained on Natural Questions [45] and SQuAD [] datasets.	Supports user-written natural questions, returns a ranked list of matching articles with highlighted answer spans. Entities in document text are also linked to external databases.
S26	Search, QA	AUEB Covid-19 Search Engine [56]	AUEB’s NLP Group	http://cslab241.cs.aueb.gr:5000/	CORD-19, BioASQ	Uses the QA model from [56] trained on BioASQ [73] data.	Supports user-written or templated questions, returns a ranked list of matching articles with highlighted answer spans. Can restrict to sections for search, e.g. Introduction and Methods.
S27	Search, QA	CovidSearch	IEETA, University of Aveiro	http://covidsearch.web.ua.pt/	CORD-19	Uses the QA model from [1].	Supports user-written natural questions, returns a ranked list of matching articles with highlighted answer spans.
S28	Search, QA	COVID-19 Research Explorer	Google Research$^\dagger $	https://covid19-research-explorer.appspot.com/	CORD-19	–	Supports user-written natural questions, returns a ranked list of matching articles with highlighted answer spans. Can ask follow-up questions.
S29	Search, QA, summarization	CAiRE-Covid [83]	The Centre for Artificial Intelligence Research (CAiRE), HKUST	https://caire.ust.hk/covid/	CORD-19, Biomedical reviews [111]	Keyword-based retrieval of paragraphs using Anserini []. Reranking and answer selection using ensemble of BioBERT QA model [49] and generalized MRQA model [82]. Summarize answers across multiple documents abstractively with BART [51] and UniLM [26], and extractively with nearest neighbor ALBERT [47] sentence embeddings.	Supports user-written natural questions, returns a ranked list of matching articles with highlighted answer spans. Provides extractive and abstractive summary over all answer spans.
S30	Search, summarization	CORD-19 Search	Vespa$^*$	https://cord19.vespa.ai/	CORD-19	Generates summaries of papers using T5 [72]. Recommends similar papers using SPECTER paper embeddings [18].	Supports user-written keyphrase queries. Recommends similar papers.
S31	Exploration	tmCovid	Emory University	http://tmcovid.com/	Pubmed abstracts, PMC full text, PubTator annotations	–	Explore papers by entity occurrence frequencies.
S32	Exploration	COVIDExplorer	Penn State’s Coronavirus-AI Project	https://coronavirus-ai.psu.edu/database	CORD-19	Unsupervised clustering of documents with maximum modularity clustering [25]. Query matching is based on bag of words similarity between query and document clusters.	Filter papers using many interactive filters or extracted topics and keywords.
S33	Exploration	CORD-19 Topic Browser	MITRE$^*$	https://topicbrowser.c19hcc.org/	CORD-19	Topics are extracted using MITRE’s Topic Modeling Neural Toolkit (TMNT) (https://github.com/mitre/tmnt)	Explore papers using extracted topics. User can select different granularities of topics.
S34	Exploration	Topic Forest	–	http://topicforest.com/biomed/coronavirus	Exploration	Topics are extracted in an unsupervised manner using variant of SGRank [21].	Explore papers through extracted hierarchy of topics and keywords.
S35	Exploration, visualization	COVID-19 Explorer	Department of Knowledge Technologies, Joz^ef Stefan Institute	http://covid19explorer.ijs.si/	CORD-19	Keyphrases are computed with RaKUn [80]. Documents are ranked by keyphrase similarity to query.	Supports boolean queries using extracted keyphrases. Visually explore embedded keyphrases.
S36	Exploration, visualization	SemViz [92]	Laboratory for Linguistics and Computation, Brandeis University	https://www.semviz.org/	CORD-19, Blender Lab COVID-19 KG [99], Protein-protein-causal-assertions dataset	Applies semantic visualization techniques to several COVID-19 graph datasets as described in [92].	Visualizes chemical-gene, pathway-disease and protein–protein interaction KBs with evidence of mentions in papers.
S37	Exploration, visualization	VIDAR-19 [106]	Yotta Conseil (independent)	https://vidar-19.yotta-conseil.fr/	CORD-19, ICD-11	Risk factors are extracted using keyword matching and regular expressions.	Visualizes risk factors within a disease hierarchy.
S38	Exploration, visualization, KB construction	SciSight [33]	Allen Institute for AI$^*$	https://scisight.apps.allenai.org/	CORD-19, MAG	Visualizes author, citation, and entity graphs using methods described in [33]	Explore papers based on affiliation and author networks, or by extracted entities and entity co-occurrences.
S39	KB construction	AIM COVID-19 Database	AIM (by APEL)$^*$	https://covid19-help.org/database	CORD-19, PubMed	–	Data presented in tabular format tracks state of treatment and vaccine development. Displays extracted entities (drugs, phase of research, class of molecule) supported by evidence from mentioning papers.

All of the included systems facilitate search or exploration over the COVID-19 literature, though some feature more specific text understanding tasks such as summarization, QA and claim verification. To facilitate a comparison between systems, we provide the following in Table 2: (i) data used, (ii) models/methods used or implemented by each system and (iii) supported user interface features. In some cases, information is not provided or could not be found about the data or models/methods used; we have indicated this using ‘–’.

The majority of systems we document here make use of public corpora and data resources, which are easily accessible from their source. Corpora like CORD-19 and LitCovid and other commonly used data resources like ClinicalTrials.gov, UMLS and biomedical ontologies adhere to FAIR principles of Findability, Accessibility, Interoperability and Reusability [103], though some systems [e.g. CovidScholar (Row S21), DOC Search (Row S5), COVID-19 Intelligent Insight (Row S12)] leverage proprietary corpora or private annotations in addition to public datasets. Additionally, though many of these systems have transparent methods or provide source code for reproducibility, a number of systems do not, as noted by missing model descriptions in Table 2.

The rest of this section is organized as follows: we define the text mining tasks used to categorize and assess the surveyed systems and use these tasks to anchor discussion and comparison of systems described in Table 2. For each task, we (i) summarize features and methodology used by the associated systems and (ii) highlight specific systems that have taken additional steps to tailor their interface for real-world use by biomedical and clinical researchers and practitioners. Such additional steps include joining literature data with biomedical KBs used in clinical settings or adding annotations created by medical experts specifically for COVID-19-related tasks. For each mentioned system, we provide a link to its corresponding row in Table 2. 

Search systems provide search experiences in which users issue queries expressing informational needs that the system satisfies with a returned collection of relevant documents. Queries can be collections of keyphrases, similar to those supported by traditional search engines like Google or PubMed. Indexing and retrieval can be implemented using open-source tools like Anserini [109] or commercial software like Amazon Kendra (https://aws.amazon.com/kendra/) or Azure Cognitive Search (https://azure.microsoft.com/en-us/services/search/). Systems like COVID papers browser (Row S2), CoronaSearch (Row S6) and CovidScholar (Row S21) compute embeddings for queries and paper text spans (i.e. sentences or entities) and retrieve documents containing nearest-neighbor spans as results. Some systems constrain the query vocabulary to entities in a known KB (e.g. COVID-19 Navigator (Row S14) allows query terms in the form of UMLS concepts). SPIKE-CORD [88] (Row S15) supports specification of regular expression-like patterns to afford users greater control over search results.

Among these search systems, Covidex [114] (Row S1), fatcat (Row S3), DOC Search (Row S5), COVID-19 Intelligent Insight (Row S12), Covid AI-powered Search (Row S13), COVID-19 Navigator (Row S14) and CovidScholar (Row S21) integrate data from many sources, going beyond documents in CORD-19 or LitCovid to other databases such as ClinicalTrials.gov, Lens, Dimensions, documents from the WHO or CDC websites and more. Several systems also leverage external KBs for entity linking, such as Vapur (Row S8), which links to ChemProt [86], COVID-19 Navigator (Row S14) and EVIDENCEMINER (Row S16), which link to UMLS, or AWS CORD-19 Search [7] (Row S24), which uses external knowledge from the Comprehend Medical KB [8, 105]. DOC Search (Row S5) and COVID-SEE [95] (Row S19) are interesting systems that incorporate extracted PICO elements and relationships in visualization and exploration, which can be especially helpful when viewing results from clinical trial papers. 

**Exploration**-focused systems assist users with discovery and understanding of documents in a corpus. Such systems may not aim to satisfy a specific informational need but are rather used to help users understand the underlying data source; as such, their interfaces facilitate unfocused data exploration and repeated interactions. Instead of supporting arbitrary user-written queries, these systems may provide a predefined set of topics or keyphrases with which to filter the documents. Keywords or keyphrases can be extracted from documents using supervised biomedical entity extraction (e.g. ScispaCy [59] and BERN [41]) or unsupervised keyphrase extraction (e.g. SGRank [21]). Paper topics can similarly be assigned via supervised document classification, as in AWS CORD-19 Search [7] (Row S24), which classifies papers using entities in the Comprehend Medical KB or in an unsupervised manner by clustering extracted keyphrases, as in COVIDExplorer (Row S32). TopicForest (Row S34) is interesting because it makes use of a learned topic hierarchy that organizes extracted keyphrases for users, although the user interface is under-developed.

Among the systems that leverage KBs, those that use curated domain-specific KBs tend to provide a better user experience, since the entities and relations in these KBs have been vetted by domain experts. IBM Watson’s COVID-19 Navigator (Row S14, https://covid-19-navigator.mybluemix.net/), perhaps the best example of this, allows users to perform boolean queries using UMLS concepts and semantic types [9]. 

**QA** systems accept queries in the form of questions and provide extracted answer spans from documents. Most QA systems over COVID-19 literature provide both search and QA functionalities, retrieving relevant documents and surfacing answering spans. Several provide additional features such as generating summaries across answers, as in CAiRE-Covid [83] (Row S29), or the ability to ask follow-up questions, as in Google’s COVID-19 Research Explorer (Row S28). Due to a lack of abundant training data specific to COVID-19, most existing QA systems needed to bootstrap their own QA training data or are trained on non-scientific domain datasets like SQuAD [73] or smaller biomedical domain QA datasets like BioASQ [91], which may result in less performant systems. Efforts like [87] and EPIC-QA (Section 5.3) aim to change this by creating public COVID-19 QA datasets for finetuning these QA systems. 

**Summarization** systems aim to provide a condensed version of a longer piece of text. The motivation is to allow readers to derive the main points of a document without expending as much effort in reading or to provide a quick overview of a document for the reader to decide whether or not to invest more time. Two systems in Table 2 incorporate summarization components: Vespa CORD-19 Search (Row S30), which generates paper-level summaries, and CAiRE-Covid [83] (Row S29), a QA system that generates multi-document summaries across answering spans. The CAiRE-Covid system generates both extractive and abstractive summaries by aggregating information across answering spans for an input query, providing a quick, high-level overview of current research. 

**KB construction** describes systems that create KBs by extracting entities and relations from text. The KB can be used to support other goals like search or exploration, or may be the primary goal, as in the AIM COVID-19 database (Row S39), which links papers to their corresponding clinical trials and trial results. The AIM database allows users to track the state of treatment and vaccine development for COVID-19. 

**Visualization** provides a visual way to interact with and understand data. Visualizations are usually coupled with extracted KBs or citation networks and provide an alternate way to explore a corpus of scientific papers. Examples include SemViz [92] (Row S36), which focuses on exploration of the CORD-19 corpus, the Blender Lab COVID-KG and protein–protein interaction datasets and SciSight [33] (Row S38), which allows users to browse the documents in CORD-19 by author, institutional affiliation, extracted entities and network relationships. 

**Augmented** reading systems attempt to improve upon the standard reading experience of papers by providing features such as entity highlighting or within-document and between-document links, e.g. COVID-19 Intelligent Insight (Row S12) highlights extracted entities directly on a paper PDF. 

**Other tasks** may be more specialized. For claim verification, a system identifies papers containing evidence that supports or refutes a claim provided in a query. SciFact [97] (Row S22) is an example of such a system. For clinical diagnostic support, a system aims to assist healthcare providers in clinical practice, e.g. the Kahun system (Row S18) allows providers to enter patient signs and symptoms, laboratory values and medical history, and provides likely diagnostic outcomes based on known associations derived from literature and other sources. 

Several of the systems we catalog use KBs or provide tight integration with controlled vocabularies (e.g. UMLS, ICD-10) or ontologies (e.g. Gene Ontology). These systems are well positioned to integrate with other data sources that use the same shared vocabularies and to leverage the automated reasoning or inference capabilities of structured KBs. We also observe that very few text mining systems in production have a clinical focus. Those that are better integrated with clinical trial data (e.g. Covidex (Row S1), DOC Search (Row S5), COVID-19 Intelligent Insight (Row S12) and AIM COVID-19 Database (Row S39)) may provide better insights for clinicians and clinical researchers. Going forward, we expect more opportunities for integrating these systems into clinical environments, where novel diagnostic and treatment strategies identified in the literature can be quickly adapted into practice.

## Shared tasks

Shared tasks, also called challenges, are community competitions that help to promote and improve performance on important tasks and have been used with success in biomedical text mining [34]. Several shared tasks were introduced early during the COVID-19 pandemic to facilitate the evaluation of text mining systems. We briefly discuss the Kaggle CORD-19 Research Challenge, the TREC-COVID *ad hoc* retrieval challenge and the upcoming EPIC-QA challenge, a QA task over both scientific documents and consumer health documents mined from trusted governmental websites.

### Kaggle CORD-19 research challenge

For the Kaggle challenge (https://www.kaggle.com/allen-institute-for-ai/CORD-19-research-challenge), participants are asked to extract answers to key COVID-19 scientific questions from the documents in the CORD-19 corpus. Round 1 of the challenge began with nine open-ended questions on COVID-19, seeking information on transmission, diagnostics and treatment. Kaggle partnered with medical experts to identify the most useful contributions from the more than 500 submissions it received.

Round 2 was designed based on this feedback and focuses on the task of table completion. Medical experts define a unique tabular schema for each question from Round 1, and participants are asked to complete the table by extracting information from CORD-19 documents. For example, extractions for risk factors should include disease severity and fatality metrics, while extractions for incubation should include time ranges. Sufficient knowledge of COVID-19 is necessary to define these schema and to understand which fields are important to include (and exclude). An example submission is described in [58]. The table completion task is somewhat analogous to extracting evidence for a systematic review, which we discuss in greater detail in Section on “Systematic review automation”.

Upon the completion of the Kaggle challenge, the community has moved towards repurposing the submitted contributions. Among the contributions are output review tables from Round 2, which provide a useful overview of research findings(https://www.kaggle.com/covid-19-contributions). Table results have been used to quickly bootstrap QA datasets [48, 87], which will be useful for training COVID-19 QA systems. Early COVID-19 QA systems rely on either existing biomedical QA datasets that do not contain questions specific to COVID-19 (e.g. BioASQ) or had to bootstrap their own COVID-19 training data through expert annotation, which is expensive and results in small-scale data. These new QA datasets and shared tasks like EPIC-QA (Section 5.3) aim to address the lack of domain-specific QA training data.

### TREC-COVID document retrieval challenge

The TREC-COVID (https://ir.nist.gov/covidSubmit/index.html) shared task [77], co-located at the 2020 Text REtrieval Conference (TREC), evaluates the ability of systems to retrieve and rank papers in CORD-19 based on their relevance to a set of pre-defined COVID-19 topics. Questions (called topics) are sourced from naturally occurring sources, such as MedlinePlus searches, Twitter, library system at Oregon Health & Science University (OHSU) and conversations with researchers. There have been five rounds of submissions and evaluations, each round introducing new topics and each anchored to a different version of CORD-19, reflecting real-world information changes as new papers are published. Round 1 began with general questions such as, ‘What is the origin of COVID-19?’, and topics have become more focused in later rounds, e.g. ‘What are the observed mutations in the SARS-CoV-2 genome?’ (Round 3) and ‘What is known about an mRNA vaccine for the SARS-CoV-2 virus?’ (Round 5).

To assess submissions for quality, task organizers recruited 60+ medical experts, including medical students from OHSU and the University of Texas Health Science Center at Houston (UTHealth) and indexers from the NLM. Top submission results are ranked by assessors to produce a partial gold ranking over the input documents.

Over 55 teams participated in the TREC-COVID challenge, including several systems from Table 2; preliminary results are presented in [96]. The results of the TREC-COVID challenge provide a ranking of retrieval systems, identifying optimal techniques for further development. Additionally, TREC-COVID topics, document sets and annotations are released for all rounds and can be leveraged to train and build retrieval systems in response to future epidemics.

### Epidemic question answering challenge

The Epidemic question answering (EPIC-QA, https://bionlp.nlm.nih.gov/epic_qa/) challenge, co-located at the 2020 Text Analysis Conference (TAC), motivates the development of QA systems for COVID-19. EPIC-QA focuses on QA over both scientific literature (supplied by the CORD-19 corpus) as well as a corpus of consumer-friendly documents derived from authoritative public-facing sites such as the website of the CDC and various agencies of the NIH.

The task is more fine-grained than TREC-COVID in that EPIC-QA evaluates system ability to extract and rank spans of text from documents, rather than full documents, that serve as answers to questions. The evaluation also judges answer comprehensiveness using a ‘nugget’-based evaluation for QA [52]. The CORD-19 collection used for EPIC-QA is the same collection used in Round 4 of the TREC-COVID challenge to enable reuse of document-level annotations curated at that time.

## Systematic review automation

**Fig. 2.**
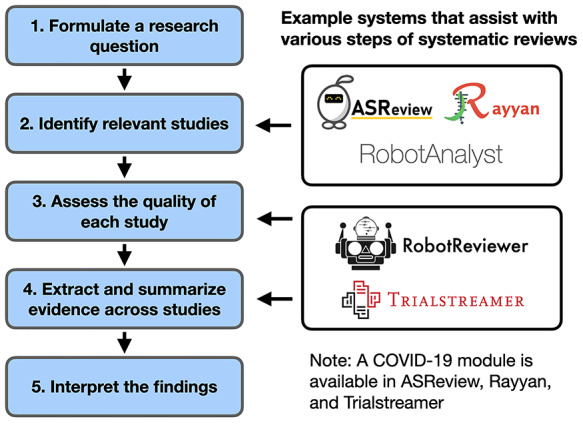
The process of systematic review construction (*left*) and example systems that assist with several steps (*right*).

Systematic reviews aim to synthesize results over all relevant published studies on a topic, providing the highest quality of evidence and recommendations for clinical and public health decisions. They have become a fixture in the biomedical literature, with many established protocol around their registration, production, publication and update [10, 15, 81]. We refer to them here because the systematic review framework is useful to keep in mind when discussing evidence summary and information overload. In Figure 2, we show the steps of systematic review construction [38]. Indeed, many of the text mining tasks we discuss previously can be framed in the context of systematic review construction. For example, search and QA can help to identify relevant documents and spans of text, table completion helps to extract structured evidence from different studies and multi-document summarization is a way of aggregating evidence across studies.

Systematic reviews have played an important role in the scientific response to COVID-19. Rapid reviews, which condense and shorten the typically months- or years-long systematic review process [39, 89], have been common. For example, rapid reviews have been published addressing research questions on infection and mortality rates [31], clinical characteristics in different subpopulations [27, 32, 65], symptoms of disease [66, 84], drug repurposing [78], COVID-19 management policies [108], as well as interactions between COVID-19 and other diseases and comorbities [20, 69, 112, 113]. Due to the large number of COVID-19 reviews, numbering in the thousands, the ones we have chosen to cite here are ones that use COVID-19 corpora like CORD-19 or LitCovid as a source of studies in addition to traditional databases like PubMed.

As the number of publications on COVID-19 has grown, it becomes increasingly difficult and expensive to produce and update these reviews. Systems that assist with or automate parts of the review process are needed. Several existing systems focus on automating parts of the systematic review process more broadly [90]. These systems focus on supporting the identification of relevant studies [5, 64, 70, 71, 94] or extracting PICO elements [22, 42, 55, 62]. The recently released Trialstreamer system allows users to discover new clinical trials using PICO-based search [61]. ASReview [5, 94], Rayyan [64] and Trialstreamer [61] all have COVID-19 modules that allow users to focus exclusively on COVID-19 papers.

The processes around creating systematic reviews have matured over the past several decades. Reviews provide trusted evidence to clinicians and policymakers and are useful for addressing information overload, as they survey and summarize information across numerous studies. Targeted methods and systems that assist in or automate systematic reviews for COVID-19 could be very impactful going forward.

## Discussion

From the start of the COVID-19 pandemic in late 2019 to now, the community has introduced numerous text mining resources and systems aimed at handling the tidal wave of the new COVID-19 literature. Over this time, we have iterated through many versions of corpora, models, systems and shared tasks. Though significant progress has been made, many open questions remain. We summarize some learnings and challenges below.

- It is helpful to have a centralized corpus of documents, such as CORD-19 or LitCovid, that is maintained and updated regularly. The existence of these corpora free the community to focus on model and system development, encouraging faster iteration and development of novel methodology.
- Intermediate infrastructure for sharing both automatically and manually produced data annotations, such as PubTator or PubAnnotation, increase the reach of annotation efforts. Annotations shared through these platforms can be reused by many downstream applications.
- Community shared tasks can be used to pool resources for evaluation and provide expert assessments on the performance of different systems. For COVID-19, the rapid submission and assessment cycles employed by tasks like Kaggle and TREC-COVID emulate the realistic challenges of rapid system development and deployment. These realistic sensibilities, though challenging to implement for organizers, may result in more robust systems that can adapt quickly to changing data and user needs.
- It is important to engage expert communities early and often, to keep the focus on real-world tasks and user needs. Tasks should be selected to maximize their similarity to relevant workflows, e.g. paper search, or systematic review construction. Because these existing workflows are validated and known to be useful, anchoring shared tasks to these workflows is more likely to result in effective systems.

Though much of the infrastructure discussed in this review have existed for decades, the realities of COVID-19 forced us to accelerate the processes around science and research, including in the steps of dataset development, model development and deployment, evaluation and publication. Adapting to these changes has produced difficulties along the way. For example, earlier releases of the CORD-19 corpus were unstable, with formats changing from week to week as we adapted to engineering challenges and user requests. Shared tasks also had to adjust accordingly. TREC-COVID, for example, was organized in five rounds, with one week windows for submission during each round. This required very rapid turnaround from both the participants submitting system for review as well as the expert assessors, who are used to working within more relaxed time constraints.

It also takes time to identify how best to involve medical experts in assessment. For TREC-COVID, the task of *ad hoc* retrieval is well defined and has historically been recognized as a useful and important text mining task. The TREC-COVID assessments, though completed in a narrower time window than typical, were still relatively easy for the expert assessors. In the case of Kaggle, however, the first round tasks were very open-ended and submissions were correspondingly diverse and difficult to compare. Medical experts were asked to manually assess more than 500 of these submissions, which was quite time-consuming. As Kaggle converged on a more structured table completion task in Round 2, these assessments became easier and arguably a better use of expert time.

## Conclusion

Methods for text mining have matured significantly over the past few decades. With COVID-19, we have an opportunity to test these methods in the sort of time- and resource-constrained setting where automation or computational assistance may be most helpful. Preliminary results are promising. Since early March, several dozen production systems tailored to various aspects of search and retrieval have been released, two shared tasks have completed with more in progress and biomedical experts have been engaged to assess and evaluate many of the systems and tools that have been publicly deployed. Aiming to help researchers manage information overload, some systems use text mining techniques to assist with conducting rapid reviews on the COVID-19 literature. As we move forward, we encourage the community to make further developments in this area. We also remind the community to keep track of practical user needs as we develop text mining systems; though much progress has been made, significant improvements are needed to provide meaningful and actionable results in the fight against COVID-19.
